# False positives in gravitational wave campaigns: the electromagnetic perspective

**DOI:** 10.1098/rsta.2024.0120

**Published:** 2025-04-10

**Authors:** Samantha Oates

**Affiliations:** ^1^ Department of Physics, Lancaster University, Lancaster, Lancashire LA1 4YW, UK

**Keywords:** gravitational waves, optical/UV, kilonova, gamma-ray bursts, transients

## Abstract

The gamma-ray burst, 170817A, and kilonova, AT2017gfo, are so far the only secure electromagnetic (EM) counterparts to a gravitational wave (GW) signal (GW170817). Further associations are required to obtain a clear understanding of these compact binary mergers, including their formation and their contribution to the production of heavy elements in the Universe. With the fourth LIGO-Virgo-KAGRA observing run currently underway, the hunt is on to find further EM counterparts to GW signals. However, GW localizations are large, typically tens to hundreds of square degrees. Finding the EM counterpart is not an easy task, given that within these areas, there will be a number of IR/optical/UV transient sources that are detected serendipitously and that are not necessarily related to the GW. Understanding how the light from these false positives evolves with time is important to rapidly confirm or rule out their association with the GW trigger. In this review, I discuss the steps involved in searching for the EM counterpart of a GW event, the false positives and how they can be quickly ruled out and why false positives are of interest even though they are contaminants to the GW-EM community.

This article is part of the Theo Murphy meeting issue ‘Multi-messenger gravitational lensing (Part 1)’.

## 1. Introduction

The era of gravitational wave (GW) astronomy began with the detection of GW150914, a binary black hole (BBH) merger detected by LIGO [1]. Since this detection, almost a decade ago, the LIGO-Virgo consortium has performed several observing runs and the GW observatories have received several upgrades to improve sensitivity. An additional GW observatory, KAGRA, has also commenced operations. Up to the end of the third observing run (‘O3’), there have been 90 compact binary mergers, with a probability of being astrophysical in origin, $p_{astro}$, of greater than 0.5, detected by GW observatories (e.g. [2]).

These compact binary mergers include BBHs, binary neutron stars (BNS) and black hole-neutron stars (BH-NS) (e.g. [3]). Compact binary mergers may be accompanied by electromagnetic (EM) light. Detecting both EM and GW signals from a merger event is important to form a fuller picture of the astrophysical phenomena than can be otherwise be achieved from either the EM or GW signal alone (e.g. [4–6]).

BNS and BH-NS mergers are expected to be accompanied by a short gamma-ray burst^1^^,^^2^ (GRB; e.g. [17,18]) and a kilonovae (KNe); red, thermal emission, produced when the ejected material, rich in neutrons, forms heavy elements, such as lanthanides, through rapid neutron capture (r-process) nucleosynthesis (e.g. [19–34]) and subsequently decays radioactively. The emission received from the BNS or BH-NS merger depends on the merging bodies, the geometry of the system and our viewing angle [35–40]. The GRB emission is highly collimated, while the KN component is expected to be more isotropic [20,21]. Emission from the short GRB will only be detected if the viewer lies close to the axis of rotation [17,18]. While the observer’s viewing angle may also affect the colours and luminosity of the observed KN emission [37,38,40]. The KN emission strongly depends on the properties of the ejecta (e.g. mass, density and composition [35]), which in turn depends on the properties of the binary components (the type of merger, their masses and spins; [36,38,39]). Magnetic fields may also affect the observed emission, for instance, by enhancing winds [36] or through magnetic spin-down of a highly magnetized NS [41]. The fate of the system post-merger also strongly affects the expected KN emission. Different KNe are expected in the BNS scenarios where the merger directly collapses to a BH, has an intermediate phase as a super/hypermassive NS or leaves a stable NS. In the BH-NS scenario, a KN is only expected if the NS is tidally disrupted and not swallowed whole [39].

BBH mergers are not typically expected to produce EM radiation [38], but there have been predictions, e.g. [42–50]. Graham *et al*. [51] proposed ZTF19abanrhr as the optical EM counterpart, for the BBH merger, GW190521 [52]. The EM flare lasted around 50 days and peaked around 50 days after the GW trigger. The flare was a 5σ deviation from the baseline Zwicky Transient Facility (ZTF) flux that had varied by only a few per cent prior to the GW event [51]. Graham *et al*. [51] concluded that this EM flare is consistent with expectations for a kicked BBH merger in the accretion disk of an AGN.

GW170817 [4,53] was the first GW event with a secure association with EM emission, marking our entry into the era of GW-EM astronomy. Associated with this GW event was a weak short GRB 170817A detected by the Fermi and Integral satellites (e.g. [54,55]). This provided the first confirmation that short GRBs are associated with the mergers of compact objects. The GW signal was also accompanied by a bright KN (AT2017gfo; e.g. [24–34,56–69]), detected 11 hours after the GW event. Several days later, an X-ray and radio counterpart emerged, consistent with off-axis GRB afterglow emission (e.g. [70–72]). The KN was initially blue and evolved to the red. This was surprising as it was expected that the high-opacity lanthanide-rich ejecta would suppress the UV optical emission. This UV component therefore provided the first evidence for a lanthanide-poor wind [24–32,34,56,57,59,60,65,67]. The detection of the KN AT2017gfo in the galaxy NGC 4993 allowed the first application of GWs as standard sirens, measuring the Hubble constant using the distance information from the GW signal and the redshift information from the EM signal (e.g. [53,73–79]).

While detecting EM counterparts to GW events is highly important, actually finding the EM counterpart is not an easy task. If the GW event is a BNS or a BH-NS merger, we may not detect gamma-ray emission from a GRB and therefore have to rely on locating the counterpart using emission at longer wavelengths. This review will focus on the search for the IR/optical/UV EM counterpart. The error regions provided by the GW detectors are large, typically of the order of tens to thousands of square degrees [2,52,80] and the optical/UV night sky is host to a vast number of transient sources that have to be excluded. This is an issue for all types of GW searches, whether the GW signal is expected to be gravitationally lensed or not. In this review, I will discuss the search methods and processes used to identify candidate optical/UV counterparts and how we separate them from false positives, essentially other transient phenomena identified during these search processes. I will focus on the search strategies for BNS and BH-NS mergers since these mergers are expected to be associated with EM phenomena. The search for EM counterparts to BBHs will have a different strategy although many of the false positives will be the same. I will discuss the nature of the false positive events and why they can be of interest themselves. In §2, I will discuss the methods used by the GW-EM community to search GW error regions and identify candidate sources. In §3, I will discuss what the false positives are. In §4, I will discuss what we find during EM searches of GW localizations from the point of view of the Ultraviolet Optical Telescope (UVOT) [81] onboard the Neil Gehrels Swift Observatory (henceforth *Swift*) [82].

## 2. The search for electromagnetic counterparts

Gamma-ray detectors, such as *Swift* and *Fermi*, are designed to observe large areas of the sky and in the case for *Swift* to trigger optical/UV and X-ray follow-up automatically with the detection of a short GRB, which is expected to accompany a GW event shortly after the merger [4,54]. The gamma-ray sky is also much less affected by contaminants compared with the IR/optical/UV. Thus the detection of a GRB soon after the GW event, within the GW localization, would imply a high chance of the GRB-GW association. Therefore, the simplest way to locate the EM counterpart to a GW signal would be for the merger to produce a short GRB and for it to trigger the usual response of telescopes designed to detect the prompt gamma-ray emission.

For a short GRB to be produced and detected, the merger should be a BNS or BH-NS, and the Earth should lie along or close to the rotation angle of the merger. However, the opening angles of the jets are expected to be narrow, between 3 and 8° [83–86]. If the jet is characterized by an angular structure, as seen in GW170817 (e.g. [70,71,87–96]), then its prompt emission could be detected for even larger viewing angles, though the chance of the Burst Alert Telescope onboard *Swift* detecting the γ-ray emission from the short GRB resulting from the merger of a BNS or BH-NS detected by LIGO-Virgo is still very small ($\approx 0.2\;yr^{-1}$; [97]).

Therefore alternative strategies for finding the EM counterpart must be considered. One such strategy, that is currently utilized by many, is one that is optimized for the detection of the approximately isotropic KN^3^. One may also observe the GRB afterglow; however, since the jet will likely be off-axis to the observer, the afterglow emission will enter our line of sight much later (e.g. [101]). The KN component is expected to produce IR/optical/UV emission and to be detected within hours after the GW signal (e.g. [38]). The exact temporal and spectral behaviour is dependent on many parameters as described in §1. Though, the general expected behaviour can be described as the following: the emission is expected to rise within the first few hours to days and then to decay on a week timescale^4^. The emission is thermal and the colour is expected to be become redder as it evolves and may be blue initially (e.g. [38]).

The strategy used for detecting the kilonova component for IR/optical/UV ground and space-based facilities is dependent on the field of view of the instrument. For narrow field instruments (≲1 square deg), such as *Swift*/UVOT, many pointings must be used to cover the GW error region. This would likely take an unreasonable amount of time and so for many narrow field of view facilities further constraints are placed in order to prioritize regions of the sky within the GW localization. Since BNS and BH-NS mergers are expected to occur in or near galaxies, a galaxy-targeted strategy is used, whereby the GW localization and the distance of the GW trigger is convolved with a galaxy catalogue (e.g. [102–104]), such that galaxies are given a probability of hosting the GW event and those with the highest probability are observed first. These catalogues are not complete, meaning that beyond a certain distance, they may not contain all galaxies at the distance we are interested in (e.g. [105]). Further, using galaxy catalogues may provide an unreasonable number of targets to follow-up for poorly localized GW events and/or those at large distance. For facilities with larger fields of view, such as the All-Sky Automated Survey for SuperNovae (ASAS-SN), the Gravitational-wave Optical Transient Observer and the ZTF [39,106–108], the GW localizations can be observed with a reasonable number of pointings. The observing strategy will, however, also depend on many other factors, that will vary from telescope to telescope and from one GW event to another, for example the required depth of observations, scheduling constraints and the time between repeat observations.

Once a given area has been observed, candidate transient sources must be identified in the images. Wide field telescopes will likely have archival images of the night sky observed on a previous night, which cover the GW localization. In this case, difference imaging is usually performed to find transient sources. Differencing is where a template image taken on a previous night is subtracted off of the science image, leaving only sources that have faded or become brighter in the resultant difference image. For narrow field instruments, difference imaging may be performed if the field has been observed previously or if archival images are available from other facilities that can be used as templates. Otherwise, transient sources are found through source detection and comparing the brightness of the detected objects with those found in catalogues, as is the case for Swift/UVOT [109]. With either method, given the large areas observed and the rapid fading nature of the KN, these processes need to be automated to locate candidate transient sources rapidly [109,110]. These pipelines may also perform further checks on the candidate sources, such as cross-matching against solar system bodies, other near-Earth objects and high proper-motion stars. These checks can be performed using databases such as Vizier [111] and the Minor Planet Checker^5^. Known transient and variable sources may also be excluded either through checks against transient alert brokers such as Lasair [112] and the Transient Name Server^6^.

## 3. False positives

After detection and initial automated checks, a number of objects may remain and require further examination. Most, however, will be ‘false positives’, as only one can be the counterpart to the GW event (if it is detected in the first place). The first main source of false positives are image artefacts. Once these have been excluded, the remaining objects are likely astrophysical in origin, either Galactic or extragalactic in nature. In the following, we will look at the main properties of the objects that have to be considered and excluded during searches of GW fields.

### (a) Image artefacts

**Figure 1.**
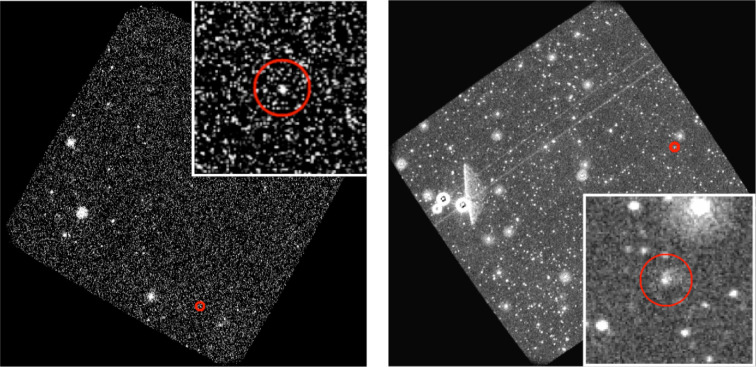
Two *Swift*/UVOT u-band images. The left panel is a confirmed astrophysical source, while the right panels show an image containing a ghost. Ghosts are scattered light features that result from secondary reflections within *Swift*/UVOT when there is a bright source in the field of view. The red circle indicates the location of the astrophysical source or ghost on the image. A zoom-in is provided in the insert. Figure reproduced from [109].

Visual inspection of the images may reveal that some of the remaining candidate sources may be artefacts produced by the optics of the telescope or as a result of imperfect image subtraction. Images may contain features such as read out streaks (vertical lines of enhanced brightness on the images produced by light from bright stars), scattered light features such as smoke rings, diffraction spikes or ghosts. Ghosts are a scattered light feature first noticed on UVOT images as a result of GW follow-up, see figure 1. They are small (a few arcsec in diameter) point-like or smudge-like sources that appear on images where there is a bright source in the field of view, which produces strong scattered light features. Ghosts are likely a result of secondary reflections within the instrument [109]. In difference images, bogus artefacts may remain due to bright star residuals, point-spread-function mismatch and/or misalignment of images [113]. Most artefacts can be ruled out quickly by eye, though some artefacts, such as the ghosts, may require more consideration.

### (b) Galactic transients

#### (i) Cataclysmic variables

Cataclysmic variables (CVs) are binary systems with a white dwarf and a companion star. CVs are found predominantly in the Galactic Plane^7^ [116,117]. The white dwarf accretes matter from the companion star. CVs can be subdivided into novae and dwarf novae based on the size of their eruptions, with novae increasing in brightness between approx. 7 and 15 magnitudes [117] and dwarf novae increasing by approx. 2 to 8 magnitudes [118,119]. CVs are also known to display flickering, minute-long changes in brightness which superimposed on the underlying behaviour (e.g. [119,120]). The brightness of the flickering can be a few millimagnitudes to more than an entire magnitude [119]. In dwarf novae, the eruption is powered by gravitational energy released during accretion [121], while in novae, thermonuclear runaway of the accreted material produces an outburst. In dwarf novae, the outbursts are semi-regular (in intervals of days to decades) and last a few days [119]. For novae, the rise time is typically 1−3 days and the outburst can last from a few days to hundreds of days [117,122]. Some novae have only been observed to erupt once, these are called classical novae. Novae outbursts are usually observed in the radio through to the optical/UV. X-ray emission can be observed simultaneously with the longer wavelength emission [123], although may not be observed until years later [114]. Novae spectra are thermal, stellar-like and evolve during the outburst. In the early stages of the nova outburst, the spectra feature broad-blue shifted absorption lines, which may have P-Cygni profiles [117], while in the later stages, emission lines dominate [124].

#### (ii) Stellar flares

Stellar flares are bursts of EM radiation produced when magnetic reconnection occurs on the surfaces of main sequence stars [125]. They occur on timescales of seconds to days, but are typically minutes long [126]. They can emit in X-ray to radio wavelengths [127]. The most common high-amplitude, fast transient found in surveys are flares from M-dwarfs [125]. M-dwarf flares are commonly detected due to the contrast of the hot (≈10000 K), blue thermal component of the flare emission with the cool (≈3000 K), red photosphere of the M-star [125]. The change in magnitude in the U-band has been measured as 0.1−5 magnitudes [128] and up to 10 magnitudes has been measured in the V-band [125]. Stellar flares are likely to be detected in crowded stellar fields and lack an underlying galaxy [129].

### (c) Extragalactic transients

#### (i) Supernovae

Supernovae (SNe) can be subdivided into two main categories depending on whether they have hydrogen emission lines in their spectra (Type II with and Type I without) [130] or based on their progenitor: core-collapse and thermonuclear (see [131] and references therein). A thermonuclear supernova, classified as Type Ia, occurs when a white dwarf in a binary system is triggered into runaway nuclear fusion either by the merger with a companion white dwarf or through the accretion of material from a companion star (e.g. [132,133]). Core-collapse SNe are the death throws of massive stars (e.g. [134]). They can be divided into Type Ib/c and Type II. Type Ib/c’s can be distinguished from Type Ia’s due to the lack of silicon absorption. Type Ic’s are separated from Type Ib due to the lack of helium emission (for a review of the differences in SN spectra, see [131] and references therein). In optical surveys, the most commonly discovered SN class are Type Ia (79%), followed by Type II (17%) and then Type Ib/c (4%), in magnitude-limited surveys [135,136]. SNe in general evolve on days to weeks timescales. They increase in brightness to a peak quickly, typically within the first two to three weeks [137], and then fade over tens to hundreds of days. Type IIs typically decline on longer timescales, compared with Type Is (e.g. [135]). Spectral energy distributions of SNe close to peak are best fit with blackbodies, which decrease in temperature with time (e.g. [138]); SNe are initially blue, becoming redder with time post-peak (e.g. [139,140]). The earliest emission that may be detected from a supernova is the shock break out. This emission is produced as the shock emerges from the stellar surface [141–146] and has been observed to rise at a rate of around 43 mag day^−1^ [145,146].

#### (ii) Tidal disruption events

A tidal disruption event (TDE) is a bright flare that arises as a consequence of a star being torn apart as it passes too close to the centre of a supermassive black hole [147–149], or potentially an intermediate black hole [150–154]. TDEs are generally found residing in the centres of galaxies, though they may be produced by black holes that are off-centre or ‘wandering’ (e.g. [155]). TDEs increase in brightness faster than they fade. They fade on timescales of tens to hundreds of days [156,157]. These timescales are longer than observed for SNe [156]. The spectral energy distributions are well fit by a blackbody with temperatures of 10^4^–10^5^ K. The temperature is approximately constant for the duration of observations [136,156]. This implies that TDEs are blue and constant in colour. SNe have been shown to have similar initial temperatures; however, SNes cool below $10^{4}\;K$ within a few weeks [138,156]. A few TDEs are shown to have relativistic jets (e.g. [158–164]). As the jet ploughs into the external medium an afterglow may arise, which produces optical/UV emission. After a few days, the emission transitions to bluer thermal emission, produced in outflows from the self-crossing shock and the accretion disk. This evolution was seen for AT2022cmc [99,164].

#### (iii) Active galactic nuclei

Active Galactic Nuclei (AGN) are supermassive black holes at the centres of galaxies that are actively accreting (e.g. [165]). AGN are variable, with the optical variability typically, ΔM≲0.5 mag [166]. However, a few AGN have exhibited a broader range in variability than previously thought. Slow-blue transients, with ΔM>1.5 mag over ≈years, have been identified [167,168]. Changing-look AGN show rapid changes in brightness, some of which have associated spectral changes [169]. There is also a recently identified population of ambiguous nuclear transients (ANTs) that have properties of both TDEs and AGN (e.g. [71,157,170,171]. Furthermore, Graham *et al*. [51] proposed the discovery of an optical EM counterpart of a BBH merger, GW190521 [52], whereby the EM emission is thought to originate from the kicked remnant of the BBH merger as it traverses the accretion disk of an AGN [49]. AGN are potentially false positives in the searches for KNe from BNS and BH-NS mergers; although note the case of GRB 191019A, which may have been a BNS that occurred in the disk of an AGN [172]. AGN may, however, be important sites to monitor for EM emission associated with BBH mergers (e.g. [42–51]).

#### (iv) Rapidly evolving transients

The cadence of optical surveys has historically been tailored to detect and follow-up Type Ia SN. However, the increase in the cadence of typical optical surveys (e.g. from 5 to 3 days; PTF versus Pan-STARRS1 [173,174]) has led to the detection of a number of rapidly evolving transients (RETs), also known as Fast Blue Optical Transients, e.g. [174–177], which includes AT2018cow (e.g. [178–180]). For these RETs, the timescales and luminosities are not easily explained by traditional SNe models [174]. RETs rise and fade more rapidly in comparison with traditional SNe. The spectral energy distributions can be fit with a blackbody with temperatures of up to $3\;\times \;10^{4}\;K$ and cool with time [175]. Several scenarios may explain these rapid events (see [177] and references therein for a list of scenarios). One possibility is that they are the result of a shock breakout and consequent shock cooling in optically thick, low-mass circumstellar wind surrounding a core collapse SN [174,175,181].

#### (v) Gamma-ray bursts

A GRB may be expected after the merger of a BNS or BH-NS that has been detected in GWs. However, there may be GRBs found in optical surveys that are not associated with the GW event. There have been a number of GRBs detected through their afterglow emission, without corresponding prompt emission. These ‘orphan’ afterglows may be orphan due to one of several reasons: the fireball is ‘dirty’, the initial Lorentz factor of the jet is below 100, and the gamma-ray emission can not escape the jet (e.g. AT2019pim^8^, [182]); an off-axis viewing angle, resulting in low-luminosity events (e.g. [183]) and an afterglow that evolves slower in comparison with an on-axis viewer (e.g. 170817A [72,88,93,96,184–186]); or simply that space-based observatories did not catch the prompt emission (e.g. [187]). Afterglows are non-thermal [188] and can be distinguished from a thermal kilonova based on colour evolution or redshift.

### (d) Excluding false positives—finding the KN

**Figure 2.**
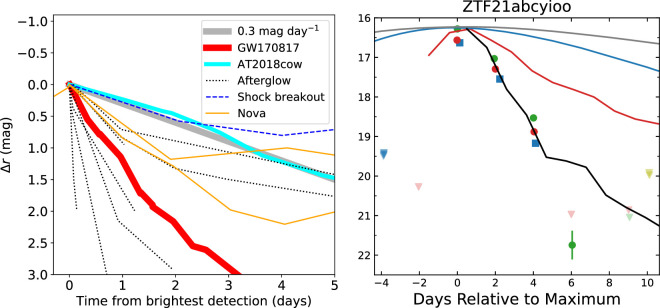
Comparison of KNe light curves with other transient sources. Left: Fig. 9 reproduced from [189], displaying the KN associated with GW170817, AT2018cow and other transient light curves found during optical transient searches with ZTF. Right: light curve of transient, ZTF21abcyioo, and several other transients, reproduced from [177]. The transient light curves are: AT2018cow (red), Type Ibn SN (blue), Type Ibc (grey) and GW170817 KN (black). Markers represent data in the following filters: ATLAS o (blue), ATLAS c (yellow), g (green) and r (red). The decay of ZTF21abcyioo is consistent with that of GW170817 KN. Investigation by [177] suggests this is a Galactic source, making it unlikely that it is a KN. Together the two panels show that KNe are among the fastest decaying optical transients and that slow evolving transients can be quickly excluded during searches for KNe. The left figure was reproduced by permission of the AAS.

Many of the objects found during the search of the GW localization will need additional spectral or photometric information to exclude them as the EM counterpart. Since spectra are costly in terms of telescope time, spectroscopy tends to be reserved for the most likely candidates, if not the KN itself once its identification is secure. Much of the candidate vetting will therefore be done with colour and temporal information obtained with photometry. With an understanding of the different types of optical transient that fill the night sky, immediate cuts can be performed. As shown in figure 2, KNe are some of the fastest decaying transients and so many objects can be excluded based on their decay rate. For instance, searches for KNe in wide optical surveys exclude objects that fade slower than 0.4 mag day^−1^, with slightly different values for different filters (≈0.6 mag day^−1^ in g-band and 0.3 mag day^−1^ in i-band) [189]. KNe are also expected to evolve quickly in colour transitioning from blue to red. If the colour of a transient remains constant with time, this would be inconsistent with expected KN behaviour. Very red transients will also be considered of interest because the blue component for some KNe may be absent; for example BH-NS mergers may predominantly produce red emission [35,38,152]. Since during the formation of BNS and BH-NS, some systems will experience large velocity kicks that will lead to their eventual merger outside of their host galaxies, further evidence would be the location of the transient. Some may be located within their host galaxies, while others may be located close to, but outside the galaxy [190–192]. One may expect the position of the merger to be similar to the projected offsets measured for short GRBs (e.g. [193,194]).

This final vetting and follow-up of candidates has to be done by human experts. However, efforts are underway to reduce the need for manual intervention during discovery and follow-up, for instance using machine learning, e.g. [195–208]. Machine learning methods require the use of training sets that guide the algorithms so they can apply knowledge to larger datasets. One of the ways to create and/or to improve a training set is to make use of citizen scientists [113]. This also has the benefit of engaging the public and enabling them to assist in scientific discovery. The best methods for classifying objects may, for now at least, be a combination of human and machine classifications [209].

## 4. EM searches in practise: the UVOT perspective from O3

As an example of the GW search process and the types of sources discovered, we now look at the results of the searches made by *Swift*/UVOT during the third LIGO-Virgo observing run.

Of the 56 public GW alerts released by the LIGO-Virgo Collaboration in O3 [52], *Swift* followed-up for 18 GW alerts following the criteria outlined in [109,210]. The LIGO-Virgo Collaboration gives a probability for each type of merger producing a given GW event. Classifying GW events by the merger types with the highest probability, the 18 GW events that *Swift* triggered are: five BBH mergers, seven BNS mergers, three BH-NS, one Mass Gap trigger, two unmodelled/burst triggers.

*Swift*/UVOT obtained 5644 unique u-band observations [109]. At the end of O3, with almost 16 years of operation, UVOT had only observed 8.4% of the sky in the u-band [109]. The UVOT GW pipeline, therefore, uses a source-detection algorithm and compares the position and brightness of detected sources with catalogued sources, rather than image differencing. A series of checks are performed by the pipeline, which include comparing the size of the major and minor axes of the source with that expected for a point source, and nearness to other bright UVOT sources and whether it is a minor body (see [109] for details).

The pipeline produces thumbnails (small image cut outs) for objects that are detected by the source-detection algorithm and that pass a quality threshold (e.g. a new source or a source at least two magnitudes brighter than a catalogued value; see [109] for full details) and are given an associated flag that represents the quality rating of the candidate; sources are flagged as Q0 if they are brighter than 19.9 mag (AB) in u and Q1 if below this value; see [109] for a full list of quality flags. The thumbnails allow the team to evaluate the reliability of possible UVOT counterparts quickly. The source-finding software sometimes misses new sources within extended sources, therefore, thumbnails are also produced for nearby galaxies to ensure no KNe are missed due to their location. Overall, the UVOT GW pipeline produced 18 459 thumbnails that were inspected visually [109]. While some of these thumbnails will be for Q0 and Q1 sources, the majority were of galaxies. During visual inspection, the majority of thumbnails were discarded; however, 27 thumbnails were found to contain ‘sources of interest’. These ‘sources of interest’ were considered to be worth investigating further as they were deemed to be astrophysical in origin and to have had a 3σ change in brightness compared with a second UVOT image (observed before or after the GW event) or archival *u*-band image from other facilities (e.g. Sloan Digital Sky Survey; SDSS [211]). For six events, we noticed a large difference in the u detection image and an archival g of at least 0.6 magnitudes. These sources did not lie close to the Galactic plane (with Galactic latitudes less than $-40^{\circ }$) and therefore are unlikely to be stars. We therefore have used the difference in the u detection image and an archival g to define these as sources of interest. Investigation of the nature of all 27 sources of interest indicates no strong evidence to identify any of the transients as counterparts to the GW events, consistent with the reports by other IR/optical/UV facilities [104,212–225].

**Figure 3.**
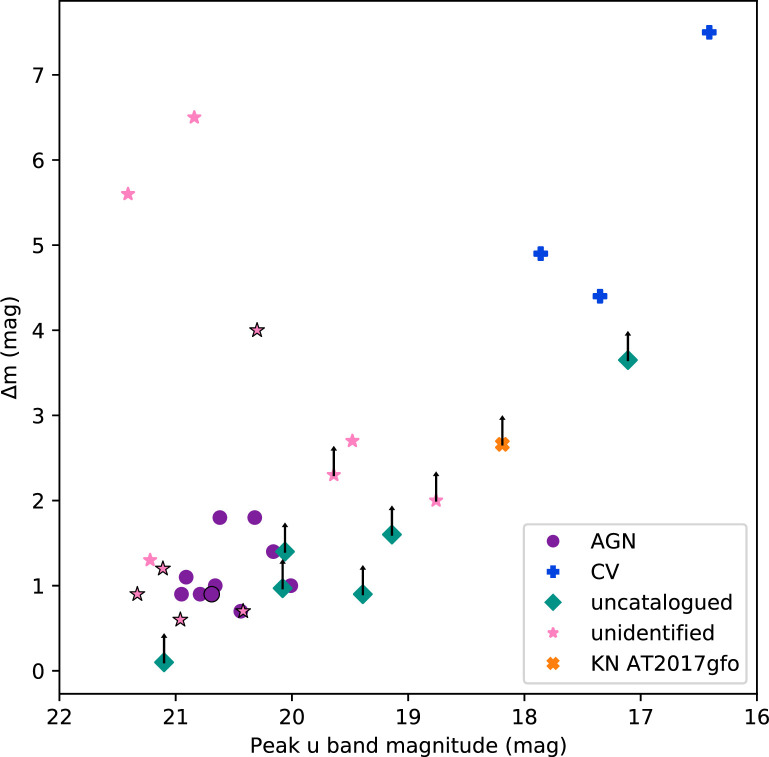
The peak u-band magnitude (AB) versus the change in magnitude, Δm for the *Swift*/UVOT sources of interest observed within the GW localizations during the third LIGO-Virgo Collaboration observing run. Figure reproduced from [109]. The sources displayed are divided into the categories: candidate AGN, cataclysmic variables, unidentified sources that had archival photometry and uncatalogued sources for which no archival photometry was available, see legend for colours and symbols. Δm is calculated using two UVOT u-band exposures or the UVOT detection magnitude and an archival g-band magnitude. Arrows indicate lower limits to Δm, and points with a black outline indicate those points where Δm was calculated using an archival g-band magnitude rather than a u-band image. Also displayed is the peak u-band magnitude vs Δm for AT2017gfo, the EM counterpart to GW170817.

*Swift*/UVOT followed up a further 13 sources in O3 that were discovered by other observatories. In total, 40 sources were investigated by [109]. Using catalogue information reported by VizieR, these sources were initially divided into five classifications: 11 candidate AGN/QSOs, three CVs, nine SNe, 11 unidentified sources that had archival photometry and six uncatalogued sources for which no archival photometry was available [109]. All available information, from archives and UVOT photometry, was then used to try to determine the nature of these sources. Figure 3, reproduced from [109], displays the change in magnitude (difference in the magnitude measured at the time of the GW follow-up and a second observation taken either from the archives or from a follow-up image, in u (preferably), or g-band (g for six sources)), versus their peak u-band magnitude measured at the time of the GW follow-up for the sources of interest^9^. The figure shows the sources clustering into various groups.

For the sources identified as candidate AGN, further investigation supported this initial identification [109]. This was based on redshift information (mostly photometric), morphology of the sources in SDSS observations, their archival light curve behaviour and their location on a IR colour-colour diagram created using WISE observations, taken from the ALLWISE catalogue [226].

The unidentified sources were found to have archival light curves spanning similar duration as those observed for the candidate AGN, but they typically had fewer observations. In figure 3, these sources fall into three main clusters. One group, consisting of five sources, is slightly fainter but has a similar magnitude change as the AGN. Archival colours and light curve behaviour for all but one of these sources is also consistent with the AGN, suggesting that at least four of these sources are AGN [109].

**Figure 4.**
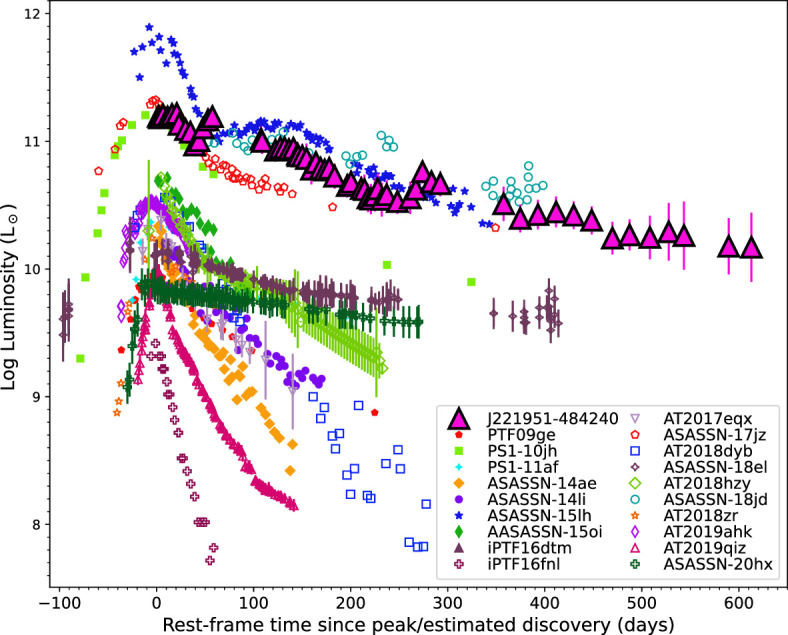
The bolometric light curve of J221951−484230 (pink triangles), reproduced from [157]. The light curve of J221951−484230 is shown together with a sample of TDEs: PTF09ge [233]; PS1−10jh [234]; PS1−11af [235]; ASASSN−14ae [236]; ASASSN−14li [237]; ASASSN−15oi [238]; iPTF16fnl [239]; AT2017eqx [240]; AT2018dyb [241]; AT2018hyz [242,243]; AT2018zr [244]; AT2019ahk [245]; AT2019qiz [246] and ANTs: ASASSN−15lh [247–250], ASASSN−18jd [251], ASASSN−17jz [170], ASASSN−18el [252], PS16dtm [253], ASASSN−20hx [254].

The second cluster of three unidentified sources are all brighter and have a larger Δm compared with the candidate AGN and in figure 3 are positioned close to KN AT2017gfo, the EM counterpart to GW170817. Two of these sources were shown to have decline rates of greater than 0.6 mag per day [109], ruling out slow evolving transients such as such as SNe [227,228], TDEs [156] and AGN [166,229], suggesting these two sources are fast evolving transients such as GRBs [188], KNe [38], fast-evolving CVs and novae (e.g. [230]), and flare stars [231,232], although the classification could not be narrowed down further [109]. The third source was investigated by [157], who determined this source, *Swift* J221951−484240, as one of the most luminous optical/UV transient sources ever recorded, see figure 4. This source was classified as an ANT. Its origin could not be determined with certainty, but it had properties consistent with a TDE and the turn-on of an AGN.

The third cluster of unidentified sources lie in the top left of figure 3, they are faint but have Δm>4. Archival observations of these sources are quite sparse, but they have faint, red archival values suggesting a faint star or distant galaxy origin. [109] suggested these were potentially fast evolving transients, similar to the two fast evolving events in the second cluster of unidentified sources.

For the six uncatalogued sources, four had very limited data and it was suggested that these sources could also be similar to the second cluster of fast evolving unidentified sources [109]. Of the remaining two sources, one (also identified as ZTF20aamvmzj and AT2020cja [220]) was found to display behaviour reminiscent of a Type II SNe. The other, which was also reported by [225] as Cand-A09, was noted to lie just beneath the CV cluster in figure 3, with only a lower limit known on Δm. It was concluded that this object is a possible CV [109].

## 5. Summary

GW localizations are currently large, typically tens to thousands of square degrees. Finding the EM counterpart is not an easy task given that within these areas there will be a number of EM transient sources that are detected serendipitously and that are not necessarily related to the GW. These are contaminants in GW searches or in other words false positives. Understanding how the light from these false positives evolves with time is important to rapidly confirm or rule out their association with the GW trigger. False positives can be classified into three main groups: instrumental artefacts (such as scattered light features or residuals in difference imaging not related to transient phenomena), Galactic and extragalactic sources. Following a GW detected BHS or BH-NS merger, a GRB and/or kilonova may be observed in the UV/optical/IR. From a geometric perspective, at least for relatively nearby GW events, the approximately isotropic kilonova is more likely to be observed than the GRB emission, which is beamed into a jet with narrow opening angles with a low probability that it is pointing in our direction. The kilonova evolves rapidly, fading beyond detection within approximately a week after detection of the GW signal. This rapid fading behaviour is inconsistent with a number of IR/optical/UV transients. This rapid temporal evolution together with the spectral evolution (becoming redder with time) is the strongest indicator that the transient may be a kilonova. ZTF21abcyioo and AT2019pim have shown that there are other sources that decay at a similar rate as AT2017gfo and are potentially false positives, and so further evidence to support a kilonova origin needs to be obtained (e.g. colour evolution, spectrum, association with a GRB occurring at a similar time and location as the GW event). Finally, sources considered as contaminants within GW searches, such as *Swift* J221951−484240, may be rare transient phenomena in their own right and of great interest to the transient community. With the fourth LIGO-Virgo-KAGRA run underway, it is an exciting time to be a member of the EM transient community, with high hopes of finding the second confirmed EM counterpart to a GW signal and in the process other rare groundbreaking transients.

## Data Availability

This article has no additional data.

## Footnotes

^1^
For a short duration gamma-ray burst, the duration time over which 90% of their gamma-ray emission (prompt emission) is measured, T90, is less than 2s [7].

^2^
The division at 2 s is rudimentary. There may be natural overlap in duration between the classes [8] and is detector dependent (e.g. [8–11]). In addition, there have been GRBs categoried by their duration as long GRBs (s), but have been shown to have other properties that suggest that they are the result of a compact binary merger event, e.g. GRB 211211A [12–14] and GRB 230307A [15,16].

^3^
In O5, GW detectors will be sensitive to events out to a distance of around 300 Mpc. Based on the behaviour of AT2017gfo, a kilonova at this distance will have a peak brightness in the r-band of ∼22 mag [98], which would still be achievable to larger facilities such as the Vera Rubin telescope [99]. For detection of GW events beyond 300 Mpc (for example the events seen face-on; see [100] and references therein), the kilonova may be too faint to be detected by most telescopes [98] and at this point the GRB may be the emission most likely to be found associated with a GW event.

^4^
This behaviour is based on theoretical expectations and observations of only two well-sampled kilonovae: to date AT2017gfo, e.g.[24–34,56–69]) and GRB 211211A [12–14]. Detections of additional kilonova may reveal a more diverse range in behaviour.

^5^

https://minorplanetcenter.net/cgi-bin/checkmp.cgi

^6^

https://www.wis-tns.org/

^7^
n.b. CVs have been observed within the local group (e.g. [114,115]).

^8^
AT2019pim was discovered serendipitously during the search of the BNS candidate GW event S190901ap [182].

^9^
We caution that for the difference in *u-g* may be due to the shape of the spectrum rather than variability and cannot be used on its own to classify these objects.
